# Supplementary material for: Phytochemical Evaluation and Anti-Inflammatory Potential of Miconia albicans (Sw.) Triana Extracts
Source: Molecules. 2022 Sep 13;27(18):5954. doi: 10.3390/molecules27185954 (PMC9500825; doi:10.3390/molecules27185954)
Supplement: Supplementary file 1 [file molecules-27-05954-s001.zip › molecules-1896273-supplementary.pdf]

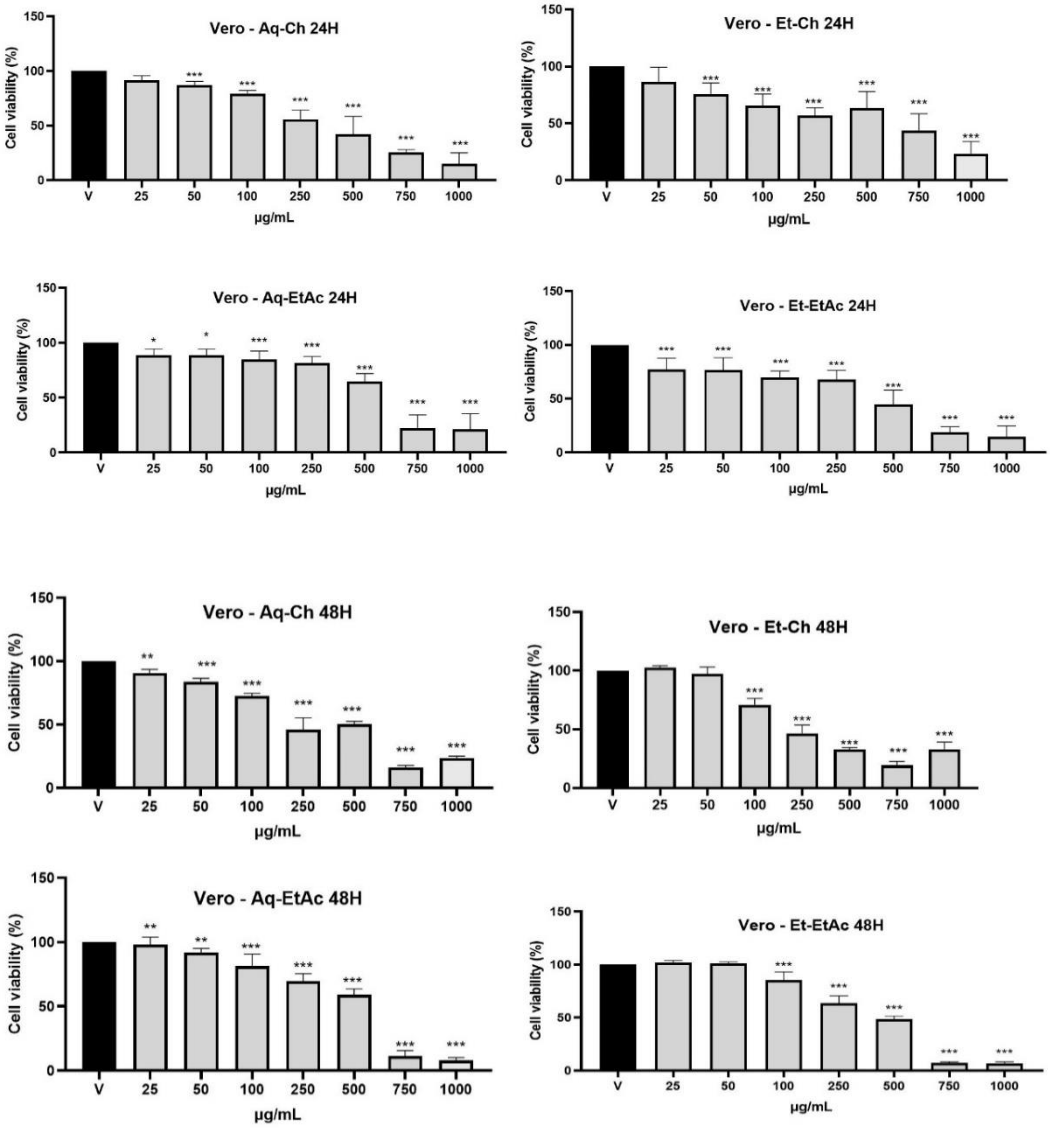

Figure S1: Cytotoxicity analysis of *M. albicans* extracts on Vero cells evaluated by PrestoBlue®. Cells were incubated with aqueous and ethanolic chloroform, and ethyl acetate fractions (25, 50, 100, 250, 500, 750 and 1000 µg/mL) for 24h and 48h. Statistical analyzes were performed by one-way analysis of variance (ANOVA). followed by Bonferroni test, selected pairs. Results represent the mean ± SD of two independent experiments (n = 2, in triplicate). *** p<0.001; **** p<0.0001 versus vehicle control (V).

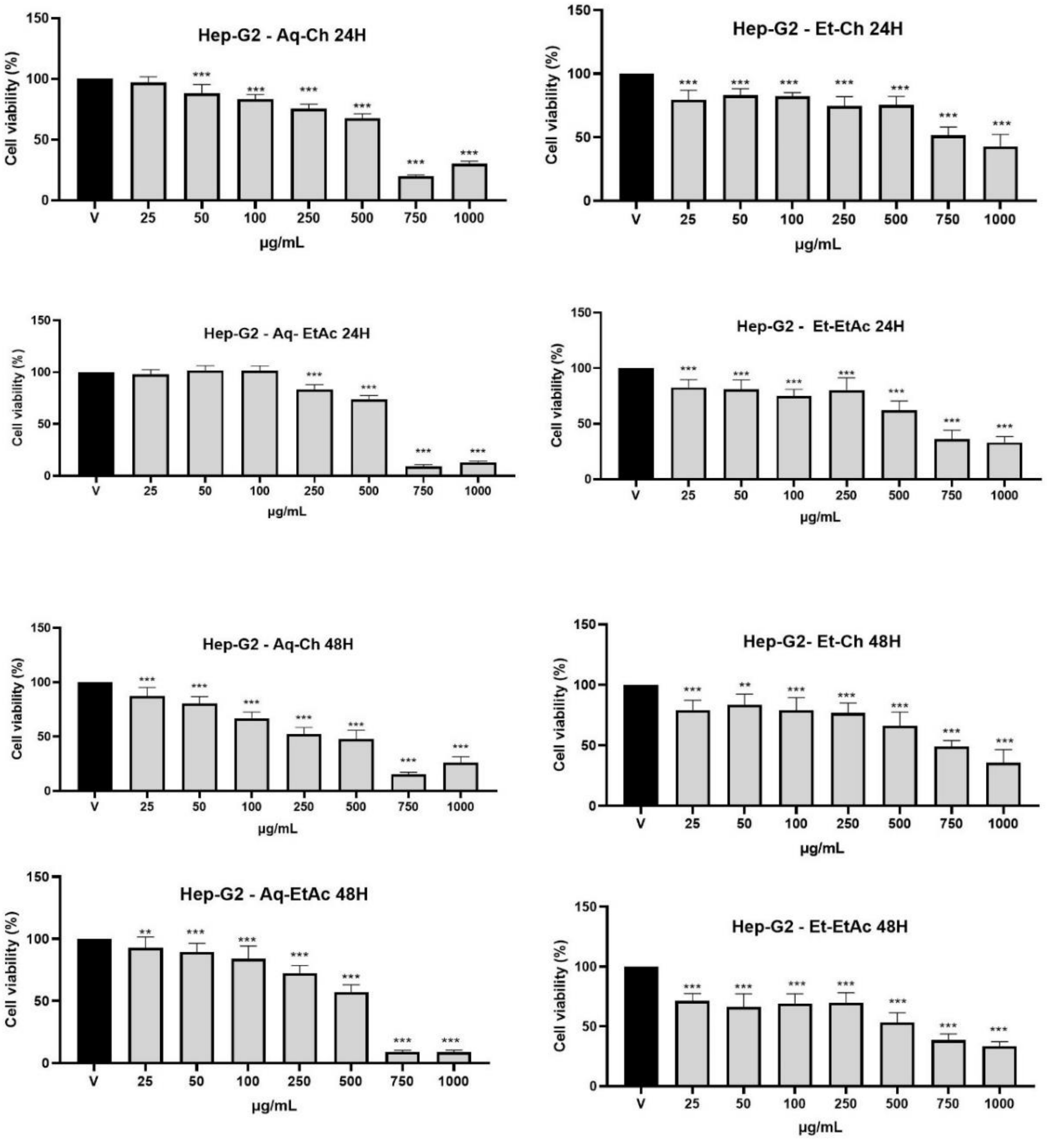

Figure S2: Cytotoxicity analysis of *M. albicans* extracts on Hep-G2 cells evaluated by PrestoBlue®. Cells were incubated with incubated with aqueous and ethanolic chloroform, and ethyl acetate fractions (25, 50, 100, 250, 500, 750 and 1000 µg/mL) for 24h and 48h. Statistical analyzes were performed by one-way analysis of variance (ANOVA). followed by Bonferroni test, selected pairs. Results represent the mean ± SD of two independent experiments (n = 2, in triplicate). *** p<0.001 versus vehicle control (V).

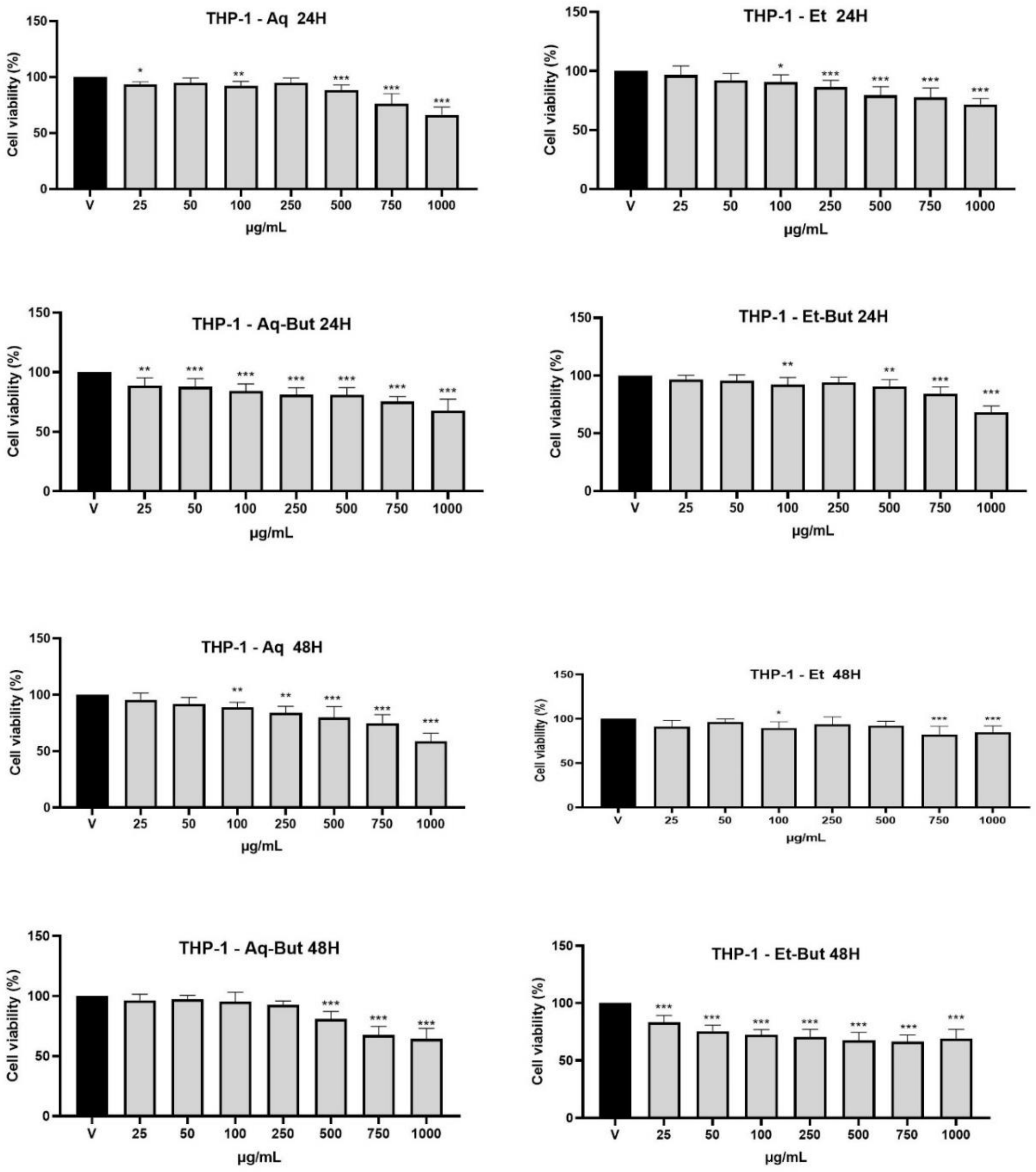

Figure S3: Cytotoxicity analysis of *M. albicans* extracts on THP-1 macrophages evaluated by PrestoBlue®. Cells were incubated with aqueous and ethanolic extracts, as well as their n-butanol fractions (25, 50, 100, 250, 500, 750 and 1000 µg/mL) for 24h and 48h. Statistical analyzes were performed by one-way analysis of variance (ANOVA). followed by Bonferroni test, selected pairs. Results represent the mean ± SD of two independent experiments (n=2, in triplicate).*** $p<0.001$ versus vehicle control (V).
